# Mechanical Characterization of Stick Insect Tarsal Attachment Fluid Using Atomic Force Microscopy (AFM)

**DOI:** 10.3390/biomimetics11010042

**Published:** 2026-01-06

**Authors:** Martin Becker, Alexander E. Kovalev, Thies H. Büscher, Stanislav N. Gorb

**Affiliations:** Department of Functional Morphology and Biomechanics, Institute of Zoology, Kiel University, Am Botanischen Garten 9, 24118 Kiel, Germany; akovalev@zoologie.uni-kiel.de (A.E.K.); tbuescher@zoologie.uni-kiel.de (T.H.B.); sgorb@zoologie.uni-kiel.de (S.N.G.)

**Keywords:** wet attachment, tarsal secretion, force measurements, nanoindentation, effective modulus, viscosity

## Abstract

Most insects secrete special fluids from their tarsal pads which are essential for the function of their attachment systems. Previous studies investigated several physical and chemical characteristics of this pad fluid in different insect species. However, there is not much known about the mechanical properties of fluid from smooth adhesive pads. In this study, we used the stress–relaxation nanoindentation method to examine the viscoelastic properties of pad fluid from *Sungaya aeta*. Force–displacement and stress–relaxation curves on single fluid droplets were recorded with an atomic force microscope (AFM) and analyzed using Johnson–Kendall–Roberts (JKR) and generalized Maxwell models for determination of effective elastic modulus (E), work of adhesion (Δγ) and dynamic viscosity (η). In addition, we used white light interferometry (WLI) to measure the maximal height of freshly acquired droplets. Our results revealed three different categories of droplets, which we named “almost inviscid”, “viscous” and “rigid”. They are presumably determined at the moment of secretion and retain their characteristics even for several days. The observed mechanical properties suggest a non-uniform composition of different droplets. These findings provide a basis for advancing our understanding about the requirements for adaptive adhesion-mediating fluids and, hence, aid in advancing technical solutions for soft or liquid temporal adhesives and gripping devices.

## 1. Introduction

Insects have lived and evolved on our planet for 400 million years. They are commonly known to show the largest diversity of all taxa within the animal kingdom [1]. One reason often used to explain this success is their ability of reversible attachment to almost any surface and, consequently, to locomote on them [2]. There are two general principles of tarsal attachment systems [3] that evolved in several groups of insects independently [4,5]. The first type are hairy systems, which are composed of a vast number of tiny setae (hairs). They can be found in flies and beetles, for example, but also in other groups of animals, like geckos and spiders [6]. The other type, smooth pads, does not involve any setae, but instead consists of highly flexible and soft cuticle [7], in contrast to the sclerotized cuticle of the exoskeleton. This type can be found, for example, in grasshoppers and bugs [3,8]. However, both types of tarsal attachment systems increase adhesion by adapting to the substrate’s profile to maximize the real contact area [9]. Furthermore, insects use an additional mechanism to enhance their attachment, by secreting a small layer of secretory fluid into the contact zone.

The phenomenon of wet attachment has gained plenty of attention from scientists during the last decades, and there have been lots of studies that examined the complex interaction of pads, substrate and fluid in various insect species [10,11,12]. The results of these studies show that a loss of fluid has strong effects on insect attachment. In addition, some studies focused more on the mechanical properties of the fluid itself, e.g., using rheology [13,14] and evaporation rate measurements [15]. Furthermore, the fluid plays a role in self-cleaning of pads, as it can fill the gaps between contaminating particles [16], and it can also affect the surface chemistry and hydrophobicity [17]. Other studies examined the chemical composition of fluid [18,19,20], and some even tested bioinspired artificial synthetic emulsions [21]. However, the influence of the adhesion fluid’s properties on the attachment abilities is not yet understood in detail [22].

Although biological surfaces are complex in general, there have been several studies which examined the attachment pads of several animal species that involve fluids, including insects and also some arachnids [10,23,24]. Referring to these studies, the attachment fluid mainly contributes to the surface conformity of the attachment pad with the substrate, enhancing the contact area on rough surfaces, but might also have further effects [11]. Another study postulated that systematic changes in the fluid’s properties might not only help to optimize attachment but could also compensate for the increasing body mass of growing stick insects. However, they found that the fluid’s properties were independent from body size [25]. Nevertheless, the mechanical properties of the solid pad material are determined by the cuticle ultrastructure. On the other hand, the properties of the fluid might be variable, based on chemical or physical composition, which probably allows further adaptation during the contact period. One previous study already showed that there is a difference in fluid viscosity in the hairy pads of beetles and flies, as well as a correlation between fluid viscosity and contact formation time during attachment [14]. This indicates that, for both examined species, there is a specific optimal value of fluid viscosity for attachment.

However, to the best of our knowledge, there have been no studies yet that directly measured the viscoelastic properties of the attachment fluid from smooth adhesive pads, as the secreted amounts of fluid from these pads were too small to use conventional methods of analysis. Despite the fact that there has been no direct analysis yet, our previous observations led to the assumption that fluid from smooth pads differs in its physical and chemical properties when compared to hairy pads. Furthermore, investigation of the frozen tarsal secretion of the stick insect *Medauroidea extradentata* (Brunner, 1907), which possesses smooth adhesive devices, revealed the presence of morphologically distinct components in the footprints of this species. It was hypothesized that these morphological differences and the range of evaporation rates represented in different fluid droplets in the secretion are a result of dissimilar physicochemical properties within the secretion [26]

In this study, we used atomic force microscopy (AFM) to examine the viscoelastic properties of tarsal fluid from the stick insect species *Sungaya aeta* Hennemann 2023, as a representative of the insects that have smooth pads. With the atomic force microscope, we were able to perform a precise analysis of local forces on the nanometer scale, which allowed for the investigation of single fluid droplets from freshly obtained footprints without the need to collect larger amounts of fluid. For this investigation, we recorded force–distance curves on single fluid droplets at different time points. Afterwards, we used the Johnson–Kendall–Roberts (JKR) model to calculate the effective modulus (E) and the surface energy from these curves, and we also investigated the dynamic viscosity (η). The aim of this study was to collect qualitative results about the viscoelastic properties of smooth pad fluids, which can be compared to our assumptions, as well as to previous findings on hairy pads.

## 2. Materials and Methods

### 2.1. Footprint Acquisition

**Figure 1:**
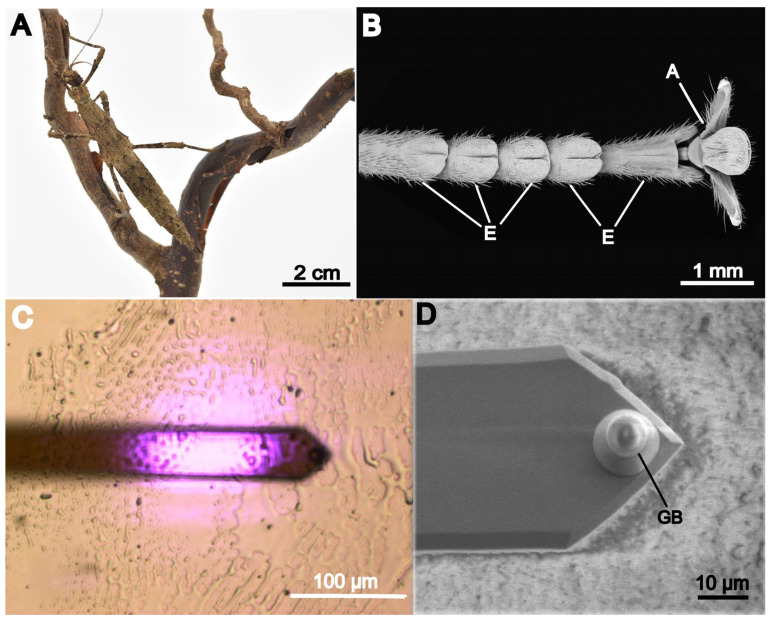
Animals and structures studied. (**A**) *Sungaya aeta* adult female. (**B**) Scanning electron microscopy (SEM) picture of tarsus from *S. aeta*, showing the attachment pads euplantulae (E) and arolium (A). (**C**) Inverse light microscopy (LM) observation of a footprint during AFM force measurements, observed from below, through the glass slide, showing the cantilever approaching the tarsal fluid droplets. (**D**) SEM picture of another cantilever tip with attached glass bead (GB) as an example.

For all experiments, only adult female individuals of stick insect species *S. aeta* (Figure 1A), originating from the lab culture of the Department of Functional Morphology and Biomechanics (Kiel University, Germany), were used. Previous studies on adhesive systems of stick insects mentioning *Sungaya inexpectata* ZOMPRO, 1996 most certainly worked on the same species (see Grote et al. 2024) [27]. The insects were kept at a natural day/night cycle and were fed with leaves of blackberry and hazel ad libitum. All individuals showed no injuries or signs of reduced health during the experiments. Footprints for force measurements were taken from random tarsi of two individuals. Therefore, one tarsus (Figure 1B) was positioned on the marked center of a glass slide, and the insect’s body was manually moved to stimulate the attachment pads for approximately 60 s. After removing the tarsus, the resulting footprint was checked first visually and afterwards in the light microscope (Figure 1C). Only footprints showing fluid droplets of more than 10 µm diameter were used for measurements. Fluid from both types of pads, arolium and euplantulae (Figure 1B), was considered.

Footprints used for white light interferometry (WLI) measurements were taken from five different individuals. For this experiment, four different treatments were performed during footprint acquisition. In the first treatment, the animal was placed on the glass slide and left unaffected for up to 6 min before removing it (1). In the second treatment, the animal was held in place with its tarsus in contact to the slide, which caused the animal to push its tarsus actively against the slide (2). In the third and fourth treatment, we gently moved the animal with our hand across the slide, with its tarsi in contact either in pushing (3) or pulling (4) direction. During footprint acquisition, sequence of animal, tarsi and treatment were randomized. Again, fluid from both types of pads, arolium and euplantulae (Figure 1B), was considered.

### 2.2. White Light Interferometry

Height measurements of freshly acquired fluid droplets were carried out with the white light interferometer (Zygo NewView 6000, Zygo Corporation, Middlefield, CT, USA) in combination with the software MetroPro (version 8.3.5, Zygo Corporation, Middlefield, CT, USA), using the 5× and 20× objectives. Height profiles were taken to measure the maximal height of the fluid droplets in each selected region.

### 2.3. Cantilever Preparation

For fluid examination, a tipless cantilever with a spring constant of 0.1046 N/m (CSC38/tipless/No Al, 0.03–0.09 N/m, µmasch, Sofia, Bulgaria) was used. The spring constant was obtained using the well-established thermal noise method. The cantilever tip was supplemented by the glass bead (Borosilicate Glass Microspheres, 7.9 ± 0.8 µm diameter, Duke Scientific Corporation, Palo Alto, CA, USA) attached to it by employing epoxy glue (UHU Plus Schnellfest, 2-k-epoxy-glue, Uhu GmbH & CokG, Bühl, Germany). The bead’s actual size was measured using LM images of the attached bead and LM images of a scaling microscopy slide as a reference, giving a radius of 5.092 µm. Prior to the measurements, the sensitivity of the cantilever was calibrated 10 times at each of 16 different positions on a glass slide, using a setpoint of 5 V. Then, the mean value acquired from this calibration was used. In addition, we took scanning electron microscope images of a prepared cantilever using a Hitachi S 4800 scanning electron microscope (Hitachi High Technology, Tokyo, Japan) to visualize the true shape of the glass bead (Figure 1D).

### 2.4. Force Measurements

All measurements were carried out with the atomic force microscope (AFM) (NanoWizard, A0016, Bruker Nano GmbH, Berlin, Germany), using the NanoWizard Control Software (version 4, Bruker Nano GmbH, Berlin, Germany). For visual control, the AFM was combined with an inverse light microscope (Axiovert 135, Zeiss, Oberkochen, Germany), equipped with a camera (Imaging Source, DFK 31AF03, Bremen, Germany). Visual observation was carried out with 20× objective. During each measurement, the cantilever indented a droplet and was then kept at constant height for 3 s before retraction. We chose a setpoint force of 10 nN, a Z-range of 10 µm and an approach speed of 10 µm/s. The AFM head motion in the z-direction was performed in a closed-loop operation.

**Figure 2:**
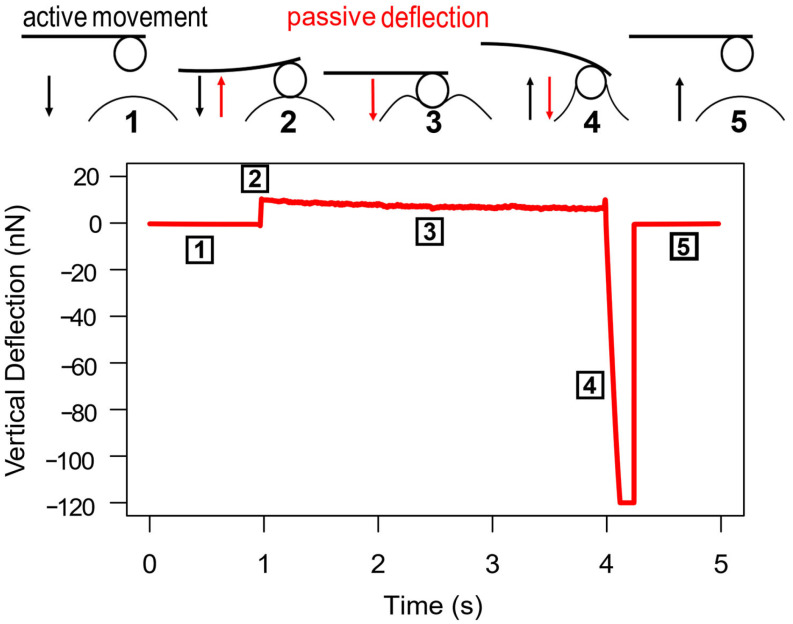
Measurement cycle on “viscous” droplet with reference to the resulting force–time curve. (1) Cantilever approaching droplet. (2) First deflection after contact with droplet’s surface until setpoint force is reached. (3) Delay phase: Stop of cantilever movement and passive deflection caused by droplet deformation. (4) Cantilever retraction and deflection caused by adhesion. (5) Contact breakage and return to the equilibrium position. Black arrows indicate active cantilever movement, red arrows indicate passive deflection without AFM head motion.

In detail, each measurement started with the cantilever approaching the droplet surface. The cantilever motion continued until the setpoint force was reached. During the following delay phase, the cantilever deflected passively (no AFM head motion) because of the deformation of the droplet below. At the end of the delay phase, the cantilever was retracted, undergoing a strong pulling force because of its adhesion to the droplet, until the contact broke and the cantilever jumped back to its equilibrium position (Figure 2). After every single measurement on a droplet, the eventual fluid sticking to the cantilever was stripped off by moving the cantilever along the glass slide. This procedure was always followed by a control glass indentation prior to the next measurement. As curves recorded with a clean cantilever on mere glass always showed a highly similar adhesion within the measurable range, any deviations due to contamination could be easily detected. As soon as this control curve showed the characteristic shape as the reference curves on mere glass, the cantilever was considered clean again.

### 2.5. Data Processing

Force curves were plotted using the software JPKSPM Data Processing (version 6.1.172, Bruker Nano GmbH, Berlin, Germany). The bead radius was 5.1 µm. A Poisson’s ratio of 0.5 was used. In all curves recorded on droplets, adhesion was so strong that it exceeded the measurable range and resulted in saturation of the retraction segment of the curve (see Figure 2). A custom script written in MATLAB (version R2025a) (MathWorks) (inspired by Maugis, 1992) [28] was used to fit the initial retraction section of force–distance curves (before saturation) according to the JKR theory. Pull-off force used in the fit was estimated as follows: the saturated section of the curves was extrapolated using two sections (before and after saturation with forces in the range 104–118 nN) by 3rd order polynomial if possible and 2nd order polynomial otherwise. The polynomial minimum was considered to be a pull-off force estimation. Statistical analysis was performed using Sigma Plot 12.0 (Systat Software Inc., San José, CA, USA). The Shapiro–Wilk Test was used to test for normal distribution. Kruskal–Wallis One-Way ANOVA on Ranks and Mann–Whitney Rank-Sum Test were used for comparison of samples. Spearman Rank Order Correlation was used for correlation analysis.

## 3. Results

### 3.1. Changes over Time

For investigation of changes in the effective modulus over time, we collected force curves on 13 “viscous” droplets from nine different prints at certain time points (45 min, 60 min, 90 min and 120 min, as well as after 24 h, 48 h and 72 h). JKR analysis provided results for 7 of these 13 droplets, with changes of max. 15% to 25% during up to 48 h only in a few cases, while the majority of measured changes was negligible (Figure S1). All force curves recorded after different periods of time still showed the characteristic properties of the respective droplet category, as previously described. Apparently, the original properties still exceed any further changes within the droplets, even after 72 h. In addition, we analyzed 18 prints by comparing all measurements within the same print, regardless of which droplet was analyzed, but referring only to the time since footprint generation for up to 120 min instead (Figure S2). Following this approach, none of the three measured parameters, namely E-modulus, surface energy or dynamic viscosity, showed any significant correlation with the time since print generation within the first two hours (Spearman Rank Order Correlation test, alpha = 0.05, *p* > 0.05).

### 3.2. Droplet Dimensions

**Figure 3:**
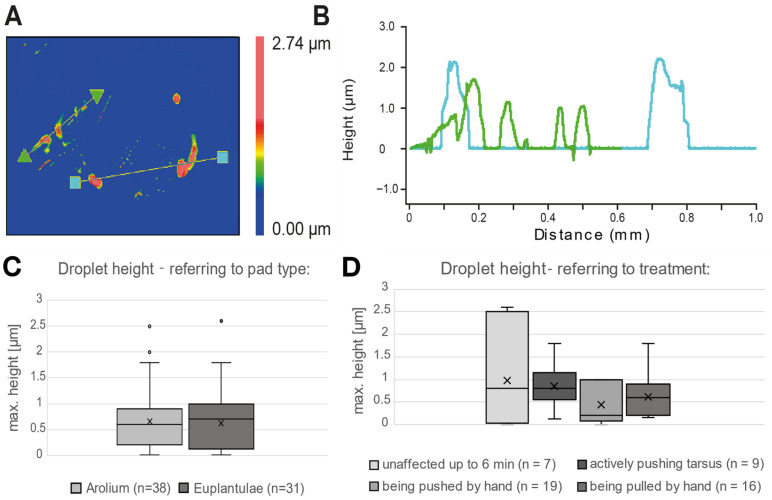
Droplet dimensions. (**A**) Example of analyzed footprint area, mapping droplet height, with two cross sections (defined by green triangles and blue squares). (**B**) Height profiles referring to the cross-sections depicted in (**A**). (**C**,**D**) Results of height measurements, with variation in droplets referring to pad type and treatments (n = sample size). Each boxplot displays the median, 25 and 75 percentiles (boxes); 10 and 90 percentiles (whiskers); and the mean is indicated by an “x” symbol.

The maximal droplet height was compared between the two types of pads, arolium and euplantulae, as well as between the treatments of the animals that were used during footprint production (Figure 3C,D). We found comparable heights for both pad types, as well as for all four treatments, with an average over all maximal height of 0.623 ± 0.561 µm. Statistical analysis revealed no significant difference between the treatments (Kruskal–Wallis One-Way ANOVA on Ranks, N1 = 19, N2 = 16, N3 = 9, N4 = 7, DF = 3, H = 3.531, *p* = 0.317). Also, between arolium and euplantulae, there was no significant difference in the height of droplets (Mann–Whitney U test, N1 = 38, N2 = 31, U = 552.5, t = 1048.5, *p* = 0.663), indicating that the maximal height of secreted droplets is independent from their origin or the used treatment.

### 3.3. Force Measurements of Different Droplets

**Figure 4:**
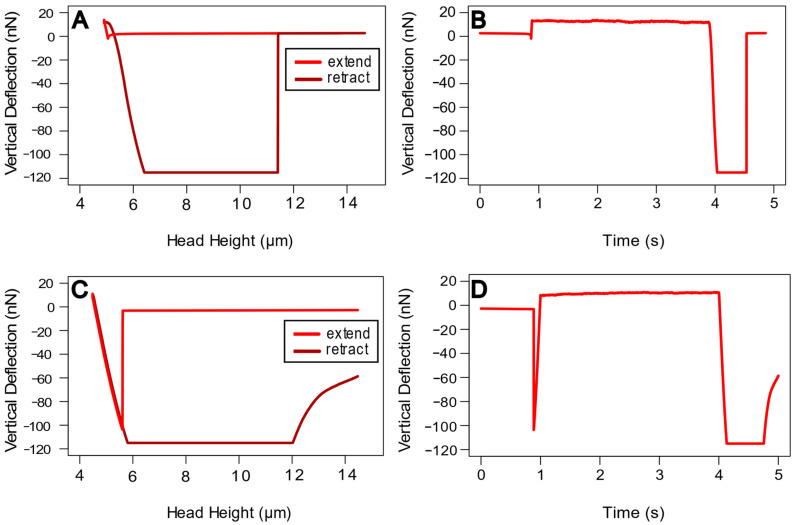
Examples of force–distance (**A**,**C**) and force–time (**B**,**D**) curves obtained via AFM. (**A**,**B**) “Rigid” droplet. (**C**,**D**) “Almost-inviscid” droplet, showing strong “jump-in” effect.

Based on the force–distance curve shapes and visual observation, the droplets were assigned to three classes: “almost inviscid”, “viscous” and “rigid”. However, the true nature of the “attachment fluid substance” still might be different than these terms suggest. Measurements on “rigid” droplets demonstrated weak jump-in force during contact formation and no distinguishable force decay during delay phase (Figure 4A,B). Visual inspection showed no deformation or other reaction in response to the cantilever indentation. “Almost inviscid” droplets demonstrated strong “jump-in” effect and no distinguishable force decay during delay phase, which means there was no measurable relaxation of the droplet during cantilever indentation (Figure 4C,D). Also, some fluid always stuck to the cantilever in this type of droplet, and so the fluid had to be stripped off after each measurement. “Viscous” droplets demonstrated characteristic relaxation during the delay phase, as shown in Figure 2 (delay phase). During this study, we analyzed 22 “almost-inviscid”, 36 “viscous” and 47 “rigid” droplets. Measurements over time revealed that all three types retained their characteristic properties even over several days. In most prints, only one of these types was observed at once. However, there were a few cases where all 3 types were found simultaneously in the same print.

### 3.4. Physical Properties of Droplets

**Figure 5:**
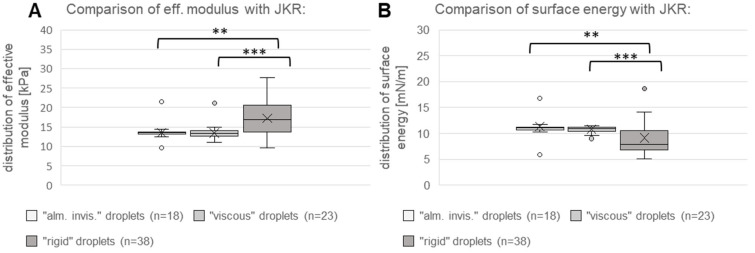
Results of force measurements with JKR model, showing the distribution of effective modulus (**A**) and surface energy (**B**) for the three types of droplets: “almost inviscid” (“alm. invis.”), “viscous” and “rigid” (n = sample size). Each boxplot displays the median, 25 and 75 percentiles (boxes); each boxplot displays the 10 and 90 percentiles (whiskers); and the mean is indicated by an “x” symbol. Significance level: ** 0.01–0.001; *** <0.001.

In total, 18 “almost inviscid” droplets, 23 “viscous” droplets and 38 “rigid” droplets were analyzed using the JKR model. For “almost-inviscid” droplets, we measured an average effective modulus of 13.569 ± 2.326 kPa, which was comparable to the result for “viscous” droplets of 13.454 ± 1.932 kPa. For “rigid” droplets, we measured a higher value of 17.258 ± 4.189 kPa. Statistical analysis showed that there was a statistically significant difference between the three groups (Kruskal–Wallis One-Way ANOVA on Ranks, N1 = 18, N2 = 23, N3 = 38, DF = 2, H = 17.382, *p* < 0.001). Pairwise comparison showed that there was no significant difference between the effective modulus of “almost-inviscid” and “viscous” droplets (Mann–Whitney U test, N1 = 18, N2 = 23, U = 184.0, t = 401.0, *p* = 0.554), while both samples were significantly different from “rigid” droplets (Mann–Whitney U test, N1 = 18, N2 = 38, U = 162.0, t = 333.0, *p* = 0.002; Mann–Whitney U test, N1 = 23, N2 = 38, U = 194.0, t = 470.0, *p* < 0.001). Furthermore, we calculated the surface energy, and the value for “almost-inviscid” droplets with 11.345 ± 2.410 mN/m and “viscous” droplets with 10.791 ± 0.633 mN/m was higher than that for “rigid” droplets with 9.128 ± 3.352 mN/m. Statistical results for surface energy were similar to those for effective modulus with an existence of significant difference for the three groups (Kruskal–Wallis One-Way ANOVA on Ranks, N1 = 18, N2 = 23, N3 = 38, DF = 2, H = 15.200, *p* < 0.001), and, respectively, a significant difference between “viscous” and “rigid” droplets (Mann–Whitney U test, N1 = 23, N2 = 38, U = 213.0, t = 937.0, *p* < 0.001), as well as “almost inviscid” and “rigid” droplets (Mann–Whitney-U test, N1 = 18, N2 = 38, U = 169.0, t = 686.0, *p* = 0.002), but not between “almost-inviscid” and “viscous” droplets (Mann–Whitney U test, N1 = 18, N2 = 23, U = 194.0, t = 391.0, *p* = 0.743). The results for effective modulus and surface energy by JKR analysis are shown in Figure 5. In addition, JKR analysis provided a result for the dynamic viscosity of all 23 analyzed “viscous” droplets with a mean value of 17.733 ± 7.226 kPa*s. A summary of all results is provided in Supplementary Material Table S1.

## 4. Discussion

### 4.1. Analysis of Viscoelastic Properties

#### 4.1.1. General Challenges of AFM Nanoindentation

The method of nanoindentation on soft biological materials comes with various challenges, as they show different behaviors than the typical materials most commonly examined with indentation methods [29,30]. Furthermore, the convenient models (e.g., Hertz model) require assumptions, including substrate homogeneity, isotropy [31,32] and low adhesion. Especially for biological materials, these assumptions often do not exactly hold due to their complex structure, composition and properties. According to our results, the tarsal fluid examined here is probably more complex and less homogenous than previously assumed [33]. As microscopy analyses of frozen tarsal fluids of another stick insect species (*Medauroidea extradentata*) have shown that the frozen tarsal secretion of stick insects has a different morphological appearance that presumably arises from the different chemical compositions of different fluid droplets [26]. There may be mixture effects of the components inside the fluid, leading to different physical properties, as we found in our results, depending on the mixture ratios. The surface properties in general can have a strong effect on the contact mechanics compared to the bulk properties of the material [34,35]; therefore, droplets with a high surface-to-volume ratio might behave differently during measurements than those with a low ratio. Furthermore, many previous studies described the thickness of the examined fluid layer and the indentation depth as other critical parameters in nanoindentation experiments. For example, Huth et al. (2019) [32] showed that the calculated effective modulus of the examined probes directly depends on the indentation depth. Another study also investigated the Hertz model for finite thickness of the examined fluid layer, leading to the conclusion that to acquire a good fit accuracy, the indentation depth should not exceed 10% of the total thickness of the fluid layer [36]. In addition, also the total thickness itself can affect the results, as for a very thin fluid layer, the interaction between the cantilever tip and the substrate below the fluid film may become dominant, resulting in an altered viscosity of the fluid between [37].

#### 4.1.2. Evaluation of the Results Using Different Contact Models

The Hertz model does not consider the adhesion between droplet and glass bead, which was even beyond the measurement range of the used cantilever (Figure 4). Thus, the elasticity moduli calculated using Hertz theory would be overestimated, and an analysis with such models as JKR required extrapolation of the force–distance curves to consider the adhesion. The effective elasticity modulus of droplets according to JKR analysis was between 13 and 17 kPa. These values of the attachment fluid elasticity modulus correspond to those for attachment structures provided by previous studies: 250 kPa up to 750 kPa for the whole pad of *Locusta migratoria* (Linnaeus 1758) [38], 27 kPa for the whole pad of *Tettigonia viridissima* (Linnaeus 1758) [8] and 125 to 188 kPa for the arolium of *Medauroidea extradentata*, while for the euplantulae of this species, higher values of 1524 to 1844 kPa were found [39]. Furthermore, the similarities and differences between the three droplet types become more pronounced, as JKR revealed a significant difference between “viscous” and “rigid” droplets. On the other hand, the similarity between “almost-inviscid” and “viscous” droplets was unexpected, considering their different behavior during measurements. It is possible that neither the cantilever nor the used model was sensitive enough to measure the true viscoelastic properties of “almost-inviscid” droplets. Also, the fitting was less precise in the case of “almost-inviscid” droplets compared to “viscous” ones. More detailed analyses in further studies are necessary to determine how strong the effective modulus of solidifying attachment fluid is correlated with its current viscosity. We previously observed that tarsal droplets from some species with smooth pads, like *S. aeta*, *L. migratoria*, etc., could not be analyzed at all with such methods as Brownian motion analysis or Interference Reflection Microscopy (IRM), since the droplets were too viscous and the collection of required amounts of fluid is cumbersome. Consequently, it is reasonable that the nanoindentation method which we used here allowed us to measure the tarsal fluid viscosity in *S. aeta*. The average dynamic viscosity estimated with a generalized Maxwell model [40] was 17.7 kPa*s. Such viscosity value further supports our conclusion that the pad fluid of *S. aeta*, and possibly fluids of smooth pads in general, is more viscous compared to the fluid of previously analyzed species. Previous studies on insect tarsal fluid described far lower values of viscosity between 10 mPa*s and 150 mPa*s for different species of flies, ants and beetles [13,14,41]. However, these studies used different methods, like Interference Reflection Microscopy (IRM) and Brownian motion measurements. The surface energy values according to the JKR model after force–distance curve extrapolation were in the range from 9 to 11 mN/m. This is far below the surface energy of water at room temperature (72.8 mN/m) and fits in the range of various polymers [42]. This rather low surface energy indicates an overall nonpolar nature of the fluid [43], which is in congruence with the results of previous studies that describe the pad fluid of stick insects as a mainly hydrophobic emulsion with an aqueous dispersed phase [33,41]. The significantly lower surface energy of “rigid” droplets might indicate an even less polar nature compared to “almost-inviscid” and “viscous” ones, which could possibly be explained by the loss of polar volatile components during hardening. It is also generally known that a higher effective modulus of a hard substance goes along with a lower surface energy, which fits well with our results (Figure 5). Furthermore, while we could not measure adhesion directly, the behavior of the cantilever during retraction can be used as a rough indication, considering whether contact breakage with the fluid happened fast, delayed or even not at all after its complete retraction (see Figure 4A,C). Thus, we found significantly higher values for E-modulus and lower values for surface energy, respectively, for curves with a fast contact breakage (N = 38) compared to curves with late breakage (N = 3) or no breakage at all (N = 55) (Kruskal–Wallis One-Way ANOVA on Ranks, N1 = 38, N2 = 3, N3 = 55, DF = 2, H = 22.796/H = 24.400, *p* < 0.001). This result fits our assumption that E-modulus should decrease and surface energy should increase with adhesion increase. However, direct measurements of adhesion would be necessary to confirm these results. As a conclusion, it can be said that analysis of force–distance curves is challenging, as liquid and hard substances have different requirements for evaluation, making viscoelastic materials, which may contain both components, even more problematic for such measurements. Hui and Baney (1998) [44] investigated the JKR theory in the context of these challenges and concluded that the model can be used for this kind of analysis but comes along with some difficulties in determination of surface energy. Nevertheless, previous studies described similar values of 26 to 27 mN/m for the congruent parameters surface tension and work of adhesion of insect tarsal fluids [9,43], in accordance with our results.

#### 4.1.3. Influence of Droplet Size and Cantilever

Based on our WLI measurements, the average maximal thickness of the secretion was 0.623 ± 0.561 µm. For AFM, only droplets of at least 10 µm in diameter were used for measurements; in most cases, they exceeded 10 µm. Therefore, all droplets were characterized as flat-shaped with a high surface-to-volume ratio. However, the strong variations in diameter and height might contribute to the variation in effective modulus and dynamic viscosity. The indentation depth was not identical between droplets due to the method. Nevertheless, the average indentation depth of force curves used for viscosity determination was approx. 60 nm. Considering the assumed average height of 0.6 µm, this falls well within the 10% limit described by Dimitriadis et al. (2002) [36]. That study also investigated the influence of the shape of the cantilever tip, leading to the conclusion that usage of sharp tips can result in overestimation of the effective modulus, while spherical tips with a radius of 2 µm or 5 µm delivered values which were comparable to macroscopic measurements. Accordingly, the bead radius that we used should have no significant influence on the results. Referring to the phenomenon of decreasing viscosity described by Darwiche et al. (2013) [37], the interaction between bead and glass slide might also have affected the results, as they found that effect already at distances of several micrometers. However, this is relativized by the fact that they used a much larger bead of 115 µm in diameter.

### 4.2. Characterization of Droplet Types

#### 4.2.1. “Almost-Inviscid” Droplets

Existence of three different droplet types demonstrating distinct characteristic force–distance indentation curves was the most intriguing result of this study. The typical properties of “almost-inviscid” droplets were a remarkable jump-in effect during contact formation, as well as absence of relaxation during the delay phase. Both features indicate that the cantilever was probably pulled down by a capillary bridge and penetrated the droplet with the bead contacting solid phase or substrate. The phenomenon of capillary bridges between a thin fluid on a flat surface and a cantilever tip has been investigated in several studies during the last decades [45,46] and depends on several factors, including tip shape and radius, as well as contact angles and even the atomic structure of the tip [47]. If some factors, like the wettability of the bead, can be compensated, it might be possible to measure even lower values of viscosity in this category of droplets.

#### 4.2.2. “Viscous” Droplets

On “viscous” droplets, viscoelastic relaxation was obviously observed during the delay phase, and the jump-in effect was much lower in contrast to fluid droplets. Several studies recorded a similar viscoelastic effect on different substrates, using AFM nanoindentation [48,49,50,51]. However, none of these substrates has been described as pure fluid. Instead, they have been described as soft materials, hydrogels and polymers, indicating that there has always been a component of macro-molecular structure from simple polymer chains up to complex cellular matrices of living cells. As the “viscous” droplets demonstrated similar rheological properties, it is likely that the tarsal fluid here consists of at least two physicochemically different compounds, as hypothesized by Thomas et al. (2023b) [26] for the tarsal secretion of *Medauroidea extradentata*.

#### 4.2.3. Time Dependence of Elastic Modulus and Viscosity

“Viscous” droplets were the only type from which we observed the effective modulus and viscosity variation after different periods of time after production. Our results revealed that both parameters show inhomogeneous changes, leading to both decrease (in most cases) and increase (in a few cases). Considering the assumed complex semi-fluid composition of this droplet type, it seems that the proportion of elastic and fluid components is changed over time in some cases. It is known that the mechanical properties of biological materials and viscoelastic materials in general depend on basic physical conditions, including time, temperature and even loading rate [52,53]. In addition, the complex composition of insect tarsal fluid [15,18] might facilitate further changes over time. External factors, like temperature and humidity, were kept as constant as possible during the measurements and should not cause any effect. Still, in general, a change in water content can affect the modulus, leading to an increase in the case of evaporation [26] or a measurable decrease in the modulus in response to increasing humidity due to water uptake, as in biopolymers like cellulose [34]. On the other hand, chemical reactions like polymerization or depolymerization can also affect the rheological properties, like the decrease in viscosity due to the enzymatic hydrolysis of soluble cellulose derivatives [54]. However, it is important to note that the measured modulus and viscosity changes over time do not lead to category change by any droplet. Therefore, one can assume that these changes are relatively small and probably do not play an important role for the character of temporal attachment and detachment events in this insect species.

#### 4.2.4. “Rigid” Droplets

The properties of the tarsal fluid become more intriguing, considering the phenomenon of “rigid” droplets. The time between footprint generation on the glass slide and the first measurement on a droplet with the AFM did not exceed three minutes, but still some droplets were already hardened. Whether this solidification process was physically or chemically based could not be deduced from the results. To the best of our knowledge, the chemical composition of the tarsal fluid from *S. aeta* has not been investigated yet. However, previous studies on the stick insect species *Carausius morosus* Brunner 1907 described the tarsal fluid of stick insects in general as an emulsion of volatile hydrophilic microdroplets in an oily continuous phase [33,41]. Emulsions in general can show complex rheological behavior depending on their composition. In this context, evaporation rate plays an important role. Previous studies have already examined some differences in composition and evaporation rate between attachment fluid from beetles and flies, showing a comparably larger fraction of volatile components in the fly fluid [15]. A follow-up study revealed that the fluids of both examined species also differed in their viscosity [14]. In the case of *S. aeta*, viscosity might also depend on the number of hydrophilic components in the fluid; however, we did not observe any detectable evaporation during the experiment in the respective droplets. While some droplets disappeared within 24 h, as could be seen using light microscopy, the “rigid” droplets retained their size even for several days. This observation is in congruence with the findings of Thomas et al. (2023b) [26] for the fluid of *M. extradentata*, which consists of components with different morphologies in the frozen state and includes droplets with different evaporation rates, also including such compounds that show no evaporation at all. In addition, the evaporation observed by Peisker and Gorb (2012) [15] happened over at least 1 h. Considering this time span, it is unlikely that the fast solidification process that we observed was caused by a loss of volatile components. Previous studies examined the chemical composition of tarsal fluid from different species, describing several components, including peptides, proteins and hydrocarbons [18,55]. One conclusion was that components like chain and branched alkenes will result in a semi-solid, grease-like consistency of the fluid [56]. However, to the best of our knowledge, a fast solidification like the one we observed has not been described for any insect species yet.

#### 4.2.5. Variation in Secretion Amount

In general, the occurrence of different droplet types was not consistent over the timeframe of the measurements, but the reasons for this remain unclear. During WLI measurements, we tested different treatments for footprint production, and although only the maximal height was measured, the results indicated that the amount of secreted fluid was independent from the treatment or particular tarsal segment. Furthermore, a previous study on *C. morosus* revealed that the tarsal secretion is independent from body size, indicating that growth or aging of the animals also has no significant effect on the key physical properties of the fluid [25]. During our study, all surrounding conditions were kept constant as far as they could be controlled. In principle, the contact with different plant surfaces can affect the attachment performance in general [57] and might also affect the tarsal secretion if the animals are able to recognize and respond to changes in the surface they encounter. Further investigations may address the question of how widespread the phenomenon of temporal adaptations among insect attachment is and which conditions can cause it.

#### 4.2.6. Possible Ecological Functions of the Fluid Complexity

It is known that the attachment structures of insects are well adapted to various kinds of surfaces. Especially in the euplantulae of stick insects, this adaptation is achieved by specific surface microstructures, which can differ strongly even between closely related species [58]. Previous studies showed that these structures are functionally relevant for the specific ecological preferences of different stick insects, as for example, smooth pads perform stronger on smooth substrates, while nubby pads are adapted to a broader range of surfaces [59,60]. More specific patterns might be an adaptation to wet substrates or specific food plants [61]. However, the role of attachment fluid has not been considered during these investigations. Thomas et al. (2023b) [26] provided results that the tarsal fluid of *M. extradentata* can be categorized into four morphologically distinct components, which presumably also differ in their viscosity due to their possible difference in chemical composition. According to these findings, the components with low viscosity produce thin films with large areas, which might contribute to the adaptation to very fine surface roughness. On the other hand, highly viscous thick films may adapt to rather coarse roughness [26].

Previous studies assumed that the complex composition of insect tarsal fluid enables fine tuning of viscosity and other properties as an additional response to various challenges which come along with the substrates in their habitat [16,17,18,26,62,63,64]. For example, the two-phasic emulsion of *C. morosus* enhances resistance against shear forces, depending on the amount of hydrophilic microdroplets [33]. The chemical composition of fluid in cockroaches comes with various functions in terms of adhesion, friction or contact-area maximization [65], and the lower viscosity of fluid in flies compared to beetles results in faster contact formation, as well as faster detachment, which might be important in order to escape predators [14]. In addition, the self-cleaning properties of both smooth and hairy pads can also be improved by the fluid, as it can wash off contaminating particles or at least fill the gaps between them to enhance the contact area [16,63]. The natural habitats of stick insects also have a great diversity of surfaces, and therefore they are well adapted to various surfaces and topographies [66]. As part of this adaptation, the properties of the attachment fluid might also be changeable in response to surrounding conditions. In a previous study, *S. aeta* was observed to be rather agile compared to another stick insect species [67]. Also during this study, the animals often showed lots of motion during footprint production, trying to escape. Nevertheless, as the movements of *S. aeta* are far slower than those of beetles or flies, a low-viscosity of tarsal fluid might be beneficial while walking on planar substrates, favoring “almost-inviscid” droplets. On the other hand, climbing on branches or leaves requires more resistance against shear forces, which could be better provided by “viscous” droplets. Finally, it is known that a semi-solid state of fluid can decrease adhesion and allow faster detachment [68]. Whether this might be the function of “rigid” droplets or not needs to be investigated in future studies.

It is known that the two types of attachment pads in stick insects have different functionality for adhesion. The arolium on each pre-tarsus is used to withstand pull-off-forces, and the euplantulae on the other tarsal segments are used to withstand shear forces [69]. Thus, it is reasonable to assume that the properties of attachment fluid differ between both types of pads. In our experiment, we were not able to ascertain the corresponding pad type for every examined print. However, we had 19 droplets that certainly originated from the arolium and 57 droplets that originated from the euplantulae. Almost all of the arolium droplets belonged to the “rigid” type, while the majority of euplantulae droplets belonged to the “viscous” type. Thus, the significant difference in effective modulus and surface energy between both droplet types also results in a significant difference between both types of attachment pads, indicating that the arolium secretes more fast-hardening liquid with high effective modulus and low surface energy. However, these results might not be representative, as we were not able to consider all droplets for this comparison. Further studies might address this question by using a true separation between both pads during footprint acquisition. We also made the observation that “rigid” droplets are easily dissolved in distilled water, indicating hydrophilic properties of at least some components of the fluid, which is in contrast to the generally assumed nonpolar nature of the attachment fluid. One previous study examined humidity as a critical factor on the properties of artificial insect-inspired fibrillar adhesive pads. The authors found that the amount of liquid secreted from the pad had a strong influence on the adhesion at low humidity, while at high humidity, additional effects, like capillary forces caused by humidity and humidity-induced softening of the pad material, strongly increased the adhesion [70]. In nature, *S. aeta* is found in moist tropical habitats [71,72]. Therefore, in a surrounding with enhanced humidity, the attachment fluid, and especially the “rigid” droplets, might show rather different properties.

## 5. Conclusions

This first attempt to analyze the mechanical properties of insect attachment fluid from smooth pads with atomic force microscopy revealed a remarkable complexity of the rheological properties of the tarsal fluid of *S. aeta*. Previous optical and macroscopic investigations on the fluid as one system did not differentiate properties caused by single subunits of this system. The qualitative analysis of the fluid indicated that the rheological properties of individual components within the secretion result in three different categories of droplets, “almost inviscid”, “viscous” and “rigid”, which tend to keep their characteristics even for several days. Thus, it is evident that the attachment fluid is a heterogenous mixture. The next step, in addition to verification and improvement of the current results, should be a detailed analysis of the chemical composition of the fluid to search for a correlation between specific components or internal reactions and the rheological properties found here. Furthermore, we were not able to analyze the adhesion properly, as we needed to extrapolate the curves. It would be great to record complete force curves, as analysis of adhesion might reveal phenomena like pull-out of material or step-wise changes. Future studies could address this question by using a stiffer cantilever. A comparison to other stick insect species might provide insights into ecological adaptability of tarsal fluids, and long-term studies in other insect species might reveal adaptive responses of their tarsal fluids to changes in environmental parameters.

One general theorem in the field of biomimetics is that biological systems have the advantage that they are multifunctional, highly adapted and optimized in millions of years of evolution. However, this comes along with a high complexity and a short lifetime compared to technical solutions [73]. Therefore, understanding these complex properties and their behavior is essential to improve their possible practical utilization. The mechanisms relying on specific wet adhesion are already used as part of bio-inspired solutions in lots of different fields [68], including medicine, for example, as part of wound treatment [74] and wearable monitoring devices [75]. Other possible applications lie in the field of bionic robots and in industrial engineering fields, such as marine anti-fouling [76] and anti-icing [77]. The examination of mechanical properties of insect tarsal fluid with AFM nanoindentation can contribute to further insights in this progress, as well as to a better understanding of the remarkable attachment abilities of insects in general.

## Data Availability

The raw data of maximal droplet height, effective E-modulus and dynamic viscosity are made available via the Supplementary Materials.

## Supplementary Materials

The following supporting information can be downloaded at https://www.mdpi.com/article/10.3390/biomimetics11010042/s1, Figures S1 and S2: Changes in effective modulus and surface energy over time. Table S1: Raw data table: height measurements, effective E-modulus and dynamic viscosity.
