# Supplementary material for: Mechanical Characterization of Stick Insect Tarsal Attachment Fluid Using Atomic Force Microscopy (AFM)
Source: Biomimetics (Basel). 2026 Jan 6;11(1):42. doi: 10.3390/biomimetics11010042 (PMC12838817; doi:10.3390/biomimetics11010042)
Supplement: Supplementary file 1 [file biomimetics-11-00042-s001.zip › Figure S1.pdf]

Supplementary graphs:

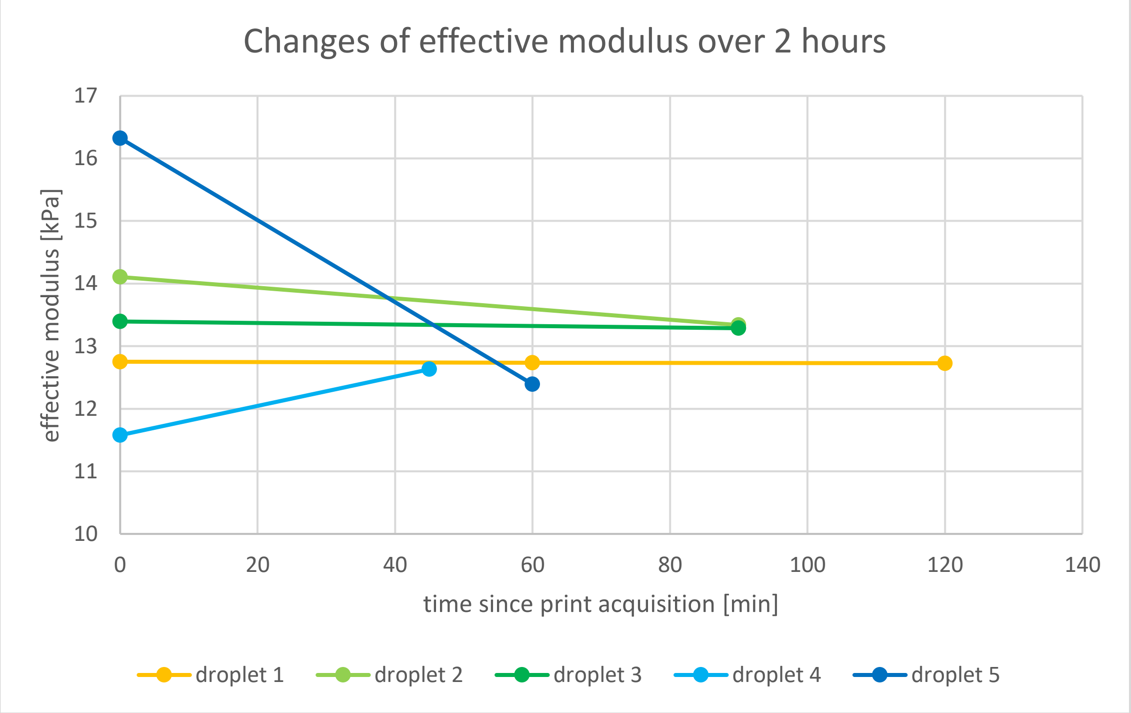

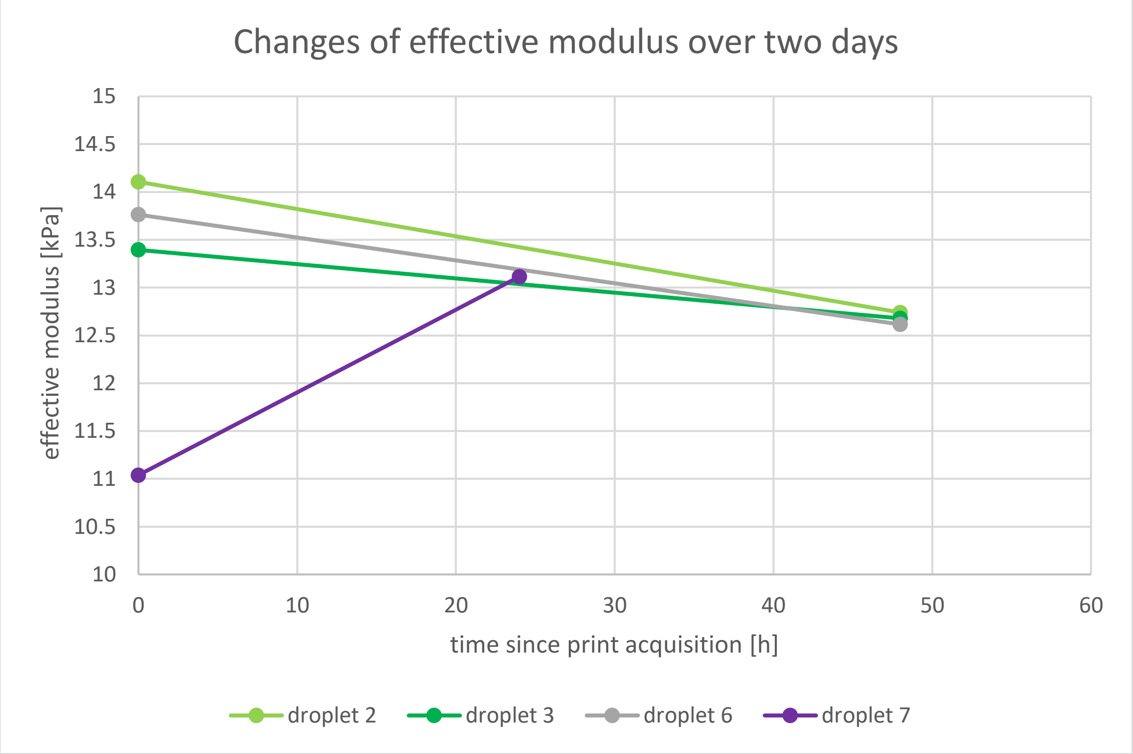

Figure S1: Analysis of changes of effective modulus on selected droplets over short and long time periods.
